# Genetic diversity and population structure of *Leishmania (Viannia) braziliensis* in the Peruvian jungle

**DOI:** 10.1371/journal.pntd.0010374

**Published:** 2022-05-23

**Authors:** Maxy B. De los Santos, Ivonne Melissa Ramírez, Jorge E. Rodríguez, Peter Beerli, Hugo O. Valdivia

**Affiliations:** 1 Department of Parasitology, U.S. Naval Medical Research Unit 6, Lima, Peru; 2 Facultad de Ciencias Biológicas, Universidad Nacional Mayor de San Marcos, Lima, Peru; 3 Unidad de Biotecnología Molecular, Universidad Peruana Cayetano Heredia, Lima, Peru; 4 Department of Scientific Computing, Florida State University, Tallahassee, Florida, United States of America; Charité University Medicine Berlin, GERMANY

## Abstract

**Background:**

Human cutaneous leishmaniasis caused by *Leishmania (Viannia) braziliensis* is highly prevalent in the Peruvian jungle, where it affects military forces deployed to fight against drug trafficking and civilian people that migrate from the highland to the lowland jungle for economic activities such as mining, agriculture, construction, and chestnut harvest.

We explored the genetic diversity and population structure of 124 *L*. *(V*.*) braziliensis* isolates collected from the highland (Junín, Cusco, and Ayacucho) and lowland Peruvian jungle (Loreto, Ucayali, and Madre de Dios). All samples were genotyped using Multilocus Microsatellite Typing (MLMT) of ten highly polymorphic markers.

**Principal findings:**

High polymorphism and genetic diversity were found in Peruvian isolates of *L*. *(V*.*) braziliensis*. Most markers are not in Hardy-Weinberg equilibrium; this deviation is most likely caused by local inbreeding, as shown by the positive *F*_*IS*_ values. Linkage Disequilibrium in subpopulations was not strong, suggesting the reproduction was not strictly clonal. Likewise, for the first time, two genetic clusters of this parasite were determined, distributed in both areas of the Peruvian jungle, which suggested a possible recent colonization event of the highland jungle from the lowland jungle.

**Conclusions:**

*L*. *(V*.*) braziliensis* exhibits considerable genetic diversity with two different clusters in the Peruvian jungle. Migration analysis suggested a colonization event between geographical areas of distribution. Although no human migration was observed at the time of sampling, earlier displacement of humans, reservoirs, or vectors could have been responsible for the parasite spread in both regions.

## Author summary

*L*. *(V*.*) braziliensis* is widespread in the Peruvian jungle region. In this region, the departments of Cusco and Madre de Dios account for a large number of patients that get infected while working in the virgin forest. For the first time, we described ample genetic diversity among Peruvian *L*. *(V*.*) braziliensis* isolates with new alleles that were not previously reported in South America. In addition, two different genetic clusters or subpopulations of *L*. *(V*.*) braziliensis* in the Amazon Rainforest of Peru were described. This finding reveals the important distribution of parasite populations and suggests a possible colonization event between ecoregions of the highland and lowland Peruvian jungle independently of recent human migration.

## Introduction

In the New World, cutaneous leishmaniasis (CL) stands out as the most common presentation of leishmaniasis, leading to disfigurement, social stigma, and functional impairment of affected patients [1].

Peru is one of the 10 countries that account for more than 75% of all CL cases reported worldwide [2]. Leishmaniasis is endemic in 19 of its 24 departments, representing more than 70% of its territory [3]. Epidemiological data indicate that between 2015–2020 Peru reported 31,463 cases of leishmaniasis, of which 92% corresponded to CL and 8% to mucosal leishmaniasis (ML) [4].

The most prevalent *Leishmania* species in the country belong to the *L*. *(Viannia)* subgenus and include *L*. *(V*.*) braziliensis*, *L*. *(V*.*) peruviana*, *L*. *(V*.*) lainsoni*, and *L*. *(V*.*) guyanensis*. Inside of Peru, the southern jungle regions of Cusco and Madre de Dios have the highest prevalence of CL, with most cases caused by *L*. *(V*.*) braziliensis* [3].

Despite the importance of leishmaniasis in Peru, there is little knowledge about local genetic diversity and population structure for *L*. *(V*.*) braziliensis* [5,6]. A study found a significant genetic diversity in 25 strains of *L*. *(V*.*) braziliensis* from Pilcopata, Cusco, Peru, co-analyzed with 99 strains from Bolivia using 12 microsatellite markers [6]. They detected a high genetic heterogeneity among local *L*. *(V*.*) braziliensis* strains, suggesting that the population is structured. Another study used 15 microsatellite markers in 31 strains of *L*. *(V*.*) braziliensis* from Brazil, Peru, and Paraguay. Only 6 of these strains correspond to Peru, and these were grouped phylogenetically with strains from the state of Acre, Brazil, and two strains of *L*. *(V*.*) peruviana* from Peru (cluster 3) [5]. Another study showed high genetic variability in 24 isolates of *L*. *(V*.*) braziliensis* using 14 microsatellite markers unrelated to *in-vitro* drug susceptibility, and clinical phenotypes; neither factorial correspondence analysis nor a phylogenetic analysis revealed geographic structure [7]

Finally, a Brazilian study that used 15 microsatellite markers and 120 strains of various *Leishmania* species, including 63 *L*. *(V*.*) braziliensis* strains, also found high genetic diversity and three subpopulations partially correlated with Brazilian geography [8]. Other studies have used amplified fragment length polymorphisms (AFLP) and whole-genome sequencing to describe *Leishmania* populations. The first found three subpopulations not linked to their geographical distribution between Peru and Brazil [9] and the second made a description of the possible geographical speciation and diversification of *L*. *(V*.*) peruviana* from populations of *L*. *(V*.*) braziliensis* that were able to cross the Andean mountain range through the Porculla pass during the late Pleistocene [10]. New studies in Peru could improve knowledge about routes of transmission, gene flow, and the role of different genetic variants in parasite virulence, infectivity, pathogenesis, and drug resistance [11–13].

In this study, we analyzed the genetic diversity and population structure of *L*. *(V*.*) braziliensis* from the Peruvian jungle region by Multilocus Microsatellite Typing (MLMT) of 10 polymorphic markers to understand the geographical distribution and population dynamics of this parasite.

## Methods

### Ethics statement

Samples were collected from patients with clinical suspicion of CL under a U.S. Naval Medical Research Unit 6 (NAMRU-6) human subjects research protocol approved by its Institutional Review Board (IRB) reference number NMRCD.2007.0018, in compliance with all applicable regulations governing the protection of human subjects. All patients participated voluntarily in the study and provided written informed consent.

### Study sites

**Fig 1:**
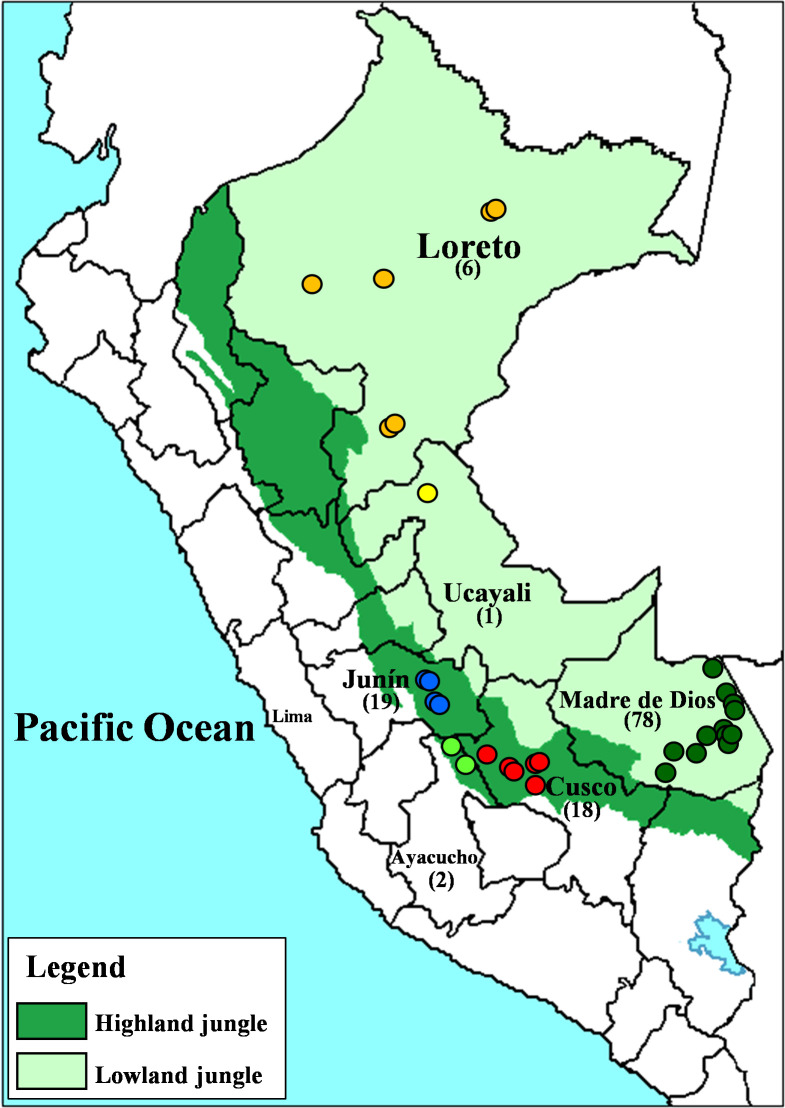
Map of Peru indicating the ecological regions of the highland jungle or *Rupa-Rupa* (dark green) and the lowland jungle or *Omagua* (light green), on which the divisions by departments are superimposed. The collection sites of the *L*. *(V*.*) braziliensis* strains are indicated by colored circles. Loreto in orange, Ucayali in yellow, Madre de Dios in dark-green, Junín in blue, Ayacucho in light-green and Cusco in red. (Modified from a map available at d-maps.com https://dmaps.com/carte.php?num_car=4765&lang=en).

Study sites comprised two ecological regions determined by different climate, geomorphology, hydrology, soil, and vegetation [14,15]: the highland jungle or *Rupa-Rupa* (400–1000 meters) that includes the departments of Ayacucho, Cusco, and Junín and the lowland jungle or *Omagua* (80–400 meters) represented by the departments of Loreto, Ucayali and Madre de Dios (Fig 1).

### Sample collection

Isolation of *Leishmania* parasites from a lesion was done as follows: a 21 G, 3 cc, 1 1/2" syringe with a needle containing 0.1 ml of sterile saline solution with 0.1 mg/mL gentamicin and 0.05 mg/ml 5-fluorocytosine was inserted horizontally or perpendicular to the internal border of the ulcer. After the needle was positioned, it was slightly rotated to the left and the right without injecting the saline. The parasite-containing tissue was aspirated and inoculated on Senekjie’s modified biphasic blood agar medium [16]. After 3–5 days, 0.1 ml of liquid phase with promastigotes was mass cultured in 5 ml of Schneider’s Drosophila medium supplemented with 30% heat-inactivated fetal bovine serum and 1% gentamicin for two or three weeks.

### DNA extraction and genotyping of samples

DNA was extracted from promastigotes of *Leishmania* strains grown in mass cultures using the DNeasy Blood and Tissue kit (QIAGEN, United States). Then, the extracted DNA was quantified using the NanoDrop ND-1000 spectrophotometer and subsequently used for the detection of species belonging to the *L*. *(Viannia)* subgenus by a kinetoplast-DNA-based PCR (kDNA-PCR) [17]. The species were determined by a FRET probes-based Nested Real-Time PCR as previously described [18].

### Multi Locus Microsatellite Typing (MLMT)

Ten highly polymorphic neutral microsatellite markers were selected from previous reports and used to determine genetic diversity indexes in the *L*. *(V*.*) braziliensis* strains: AC52, B3H, Ibh3, E11, 7GN, EMI, LBA, ARP, G09, and LRC (Table 1) [5,6,19].

**Table 1:** Characteristics of microsatellite markers used in this study.

Locus	Primer sequence (5’-3’)	Motif	*Ta*(°C)	Dye	Allele size (bp)
**Poolplexing group A**					
**B3H**	5’-GGTATGCGTGGATATGAAGC-3′	(AC)_13_	60	6-FAM	59–93
	5’-CTCGGCATCGCAGTTTC-3′				
**AC52**	5′-CATCTACGGCTGATGCAGAA-3′	(CA)_18_TA(CA)_14_	58	NED	86–140
	5′-CGTCTGGCTAAAGTGGGAAT-3′				
**Ibh3**	5′-GGAGAGGCTGCGATGTATCT-3′	(GT)_2_GG (GT)_2_GG(GT)_4_	58	HEX	117–133
	5′-CAGGGCTGTCTTGACGAAG-3′				
**Poolplexing group B**					
**E11**	5′-TGCGTAGGGCAAAGGAGTT-3′	(GA)_10_	58	6-FAM	93–111
	5′-GGGTGTCTGCCTGCATTC-3′				
**7GN**	5’-CTCCTGGAACGGCTAACAC-3′	(AC)_11_	60	HEX	105–131
	5’-TGATATGAGGCACATTCAGC-3′				
**LBA**	5′-CCTCTGTGAGAAGGCAAGGA-3′	(GA)_11_	64	HEX	165–193
	5’-GCTGCACATGCATTCTCTCGT-3′				
**EMI**	5′-CGCTGAAGCACGGCGAATG-3′	(GT)_20_	60	NED	180–206
	5′-CGTAGCTCCTCTGTCCGTTC-3′				
**Poolplexing group C**					
**LRC**	5′-CTGCCTCTGCCTCACCTACT-3′	(GT)_17_	60	HEX	112–166
	5′-CTAACCCTCACACTCCCCATC-3′				
**ARP**	5′-GGCTTCGGTCTGTTCGACTA-3′	(GT)_10_	60	NED	129–173
	5′-CACCCACTCGCATCCGTA-3′				
**G09**	5′-CAAGCAGGCAAGAGTCTGAAA-3′	(CA)_3_(GA)_12_	60	6-FAM	152–168
	5′-GTCTCCCGTATTGCTCTCTCTA-3′				

*Ta*: annealing temperature

PCR was performed in 10 μl mix containing 1X PCR Buffer (20 mM Tris-HCl pH 8.4, 50 mM KCl), 1.5 mM MgCl_2_, 200 μM dNTPs, 0.4 μM of each primer, 0.05 U/μl Platinum *Taq* DNA Polymerase (Thermo Fisher) and 0.4 ng DNA. Fluorophores in the forward primers (6-FAM, HEX, and NED) and PCR conditions were modified from the original publications to eliminate split and stutter peaks [5,6].

Amplification conditions were: initial denaturation at 94°C for 5 minutes, followed by 35 cycles of 94°C for 30 seconds, with a gradient annealing of 55°C to 65°C for each primer for 30 seconds, and 72°C for 1 minute; then a final extension step of 72°C for 15 minutes. PCRs were performed in triplicate, and DNA from *L*. *(V*.*) braziliensis* MHOM/BR/84/LTB300 reference strain was used as a positive control of amplification.

PCR products from multiple microsatellite markers were combined in a single lane, a procedure called pseudo-multiplexing or poolplexing (Table 1) depending on allele size and fluorophores wavelength emitted to avoid overlapping between loci [20]. Fluorescently labeled PCR products were separated on 50 cm electrophoresis capillaries with POP7 in a 3130xl Genetic Analyzer (Applied Biosystems) using GeneScan 350ROX dye as a size standard. Electropherograms were analyzed to determine the allele size-calling using the second-order least squares method provided by GeneMapper v4.0 [21] The reference strain for *L*. *(V*.*) braziliensis*, MHOM/BR/84/LTB300, was used in each electrophoretic run to provide a consistent size marker for each of the ten microsatellite loci [5–8]. The sizes of the identified alleles were recorded in whole numbers for the analysis with GenAlEx 6.5 [22].

### Genetic diversity indexes

We calculated the number of alleles (*N*_*A*_), observed heterozygosity (*H*_*O*_), and expected heterozygosity (*H*_*E*_) using the software GenAlEx 6.5. Allelic richness (*r*_*n*_) was calculated through the rarefaction method using FSTAT 2.9.3.2 [23], whereas polymorphic information content (PIC) measures the ability of a marker to detect polymorphisms, was calculated with CERVUS 3.0 [24]. The 95% confidence interval for inbreeding coefficient (*F_IS_*) was determined using 100,000 bootstraps for populations in FSTAT. The frequencies of null alleles, stuttering effects, and typographic errors were evaluated by Micro-Checker 2.3.3 [25]. Hardy–Weinberg deviations for each locus, fixation indices *F*_*ST*_ and *Rho*_*ST*_, considering different sample sizes among populations, were estimated with the method of Weir and Cockerham [26] using the software Genepop 4.4 [27]. Additionally, we performed the Analysis of Molecular Variance (AMOVA) with ARLEQUIN v3.5 [28] using 10,000 permutations and estimated genetic variation at different levels: 1) among groups or ecoregions, considering the highland jungle or *Rupa-Rupa*, and the lowland jungle or *Omagua*, 2) among populations within groups or Peruvian departments within ecoregions (Ayacucho, Cusco, Junín for highland jungle, Madre de Dios, Loreto and Ucayali for lowland jungle; and 3) within populations or between individuals.

### Genetic structure and population dynamics

The genetic structure of the *L*. *(V*.*) braziliensis* population was determined through Bayesian grouping analysis by STRUCTURE v2.3 [29] based on an admixture model with K varying from 1 to 10, 30 independent runs were performed for each value of K with a burn-in of 1,000,000, followed by 1,000,000 Markov Chain Monte Carlo (MCMC) iterations.

The optimal number of subpopulations was determined using the ΔK method implemented in Structure Harvester v0.6.94 [30]. We performed a factorial correspondence analysis (FCA) using GENETIX [31] to condense allelic frequencies information in factors for multidimensional visualization without a priori assumption of grouping.

Additionally, POPULATION v1.2.32 was used to generate Neighbor-Joining tree using Nei’s minimum distance (D_A_) [32] and MEGA-X to visualize the phylogenetic tree with radiation topology and circle style.

### Linkage disequilibrium analysis

Linkage disequilibrium was assessed using LinkDos [33], assuming that pairs of loci are at linkage equilibrium. The Bonferroni correction was applied to decrease type I errors [34]. Multilocus linkage disequilibrium was evaluated using the method of the standardized index of association (r̄_d_). We used the R package poppr [35] to test the null hypothesis of no linkage disequilibrium with 1000 permutations.

### Colonization history analysis of *L*. *(V*.*) braziliensis*

Different models of gene flow and colonization between lowland (L) and highland (H) jungle were evaluated and compared using a Bayesian approach of the structured coalescent implemented in MIGRATE-N v4.4.4. [36]. Each model was run using a Markov chain Monte Carlo scheme with four parallel chains (heated chains) to support the model selection approach (29). For each locus, ten replicate heated chains were used. Each replicate was burned-in for 100,000 steps and then ran for 1,000,000 steps collecting 100,000 samples every ten steps. We specified prior distributions for the parameter used in the models: the mutation-scaled population size Theta, the mutation-scaled immigration rates, and for some models the mutation-scaled divergence time used a uniform prior distribution with bounds of 0 and 100 generation times mutation rate. The units for the mutation-scaled population sizes were four times the number of individuals times mutation rate per generation, the mutation-scaled immigration rate was immigration rate per mutation rate, and the mutation-scaled divergence time was generation times per mutation rate.

The tested models were: (1) Immigration ongoing and recurrent, assuming that both populations existed for a long time: (a) Migrants only move from L to H. (b) Migrants only move from H to L. (c) Migrants move between both jungles at potentially different rates. (2) Divergence with immigration, considering one population split from the other some time ago: (a) L is ancestral, H is colonized from L, ongoing/directional gene flow L➜H. (b) H is ancestral, L is colonized from H, ongoing/recurrent gene flow between L←→H. (c) L is ancestral, H is colonized from L, ongoing/recurrent gene flow between L←→H. (d) H is ancestral, L is colonized from H, ongoing/directional gene flow H➜L. (3) Divergence without immigration, considering one population split some time ago from the other: (a) H is ancestral, L is colonized from H, no gene flow/migration afterward. (b) L is ancestral, H is colonized from L, no gene flow afterwards. (c) L is ancestral, H is colonized from L, no gene flow afterward. Models 3b and 3c are the same with different prior ranges for divergence in generation times mutation rate. The log marginal likelihoods were used to estimate the log Bayes factor, which was used to estimate the best model.

## Results

### Optimization of microsatellite markers amplification

The optimal quantity of *Leishmania* DNA was estimated in 0.4 ng (equivalent to 4.8 x 10^3^ parasites), which is 25 to 250 times less of template DNA than described in previous articles where authors used between 10 ng to 100 ng of template DNA to amplify microsatellite markers of *Leishmania* by PCR (equivalent to 1.2 x 10^5^ up to 1.2 x 10^6^ parasites) [5,6,19]. All potential artifacts were removed based on suggestions previously described [20,37]. Split peaks, stuttering or shadow bands, and false peaks were eliminated by decreasing primer concentrations in markers 7GN, AC52, ARP, B3H, EMI, and Ibh3 (0.06 μM final concentration of each primer). Optimizing thermal conditions resulted in better hybridization temperatures for most primers at 60°C, whereas AC52, E11, and Ibh3 at 58°C, and LBA at 64°C.

We eliminated fluorescence saturation signal and background noise using a 1:1000 dilution of PCR products for all markers to perform fragment analysis. This process allowed the proper allocation of alleles in most *L*. *(V*.*) braziliensis* isolates. However, no amplification in G09 (four samples) and B3H, EMI, and E11 (one sample each one) were observed (S1 Table). Micro-Checker found the presence of null alleles in all markers in the total population due to an apparent general excess of homozygotes (heterozygous deficit). Also, null alleles were apparently present in the E11 and EMI markers of cluster 1 (N = 25) and the B3H marker of cluster 2 (N = 99) (S2 Table).

### The population of *L*. *(V*.*) braziliensis* exhibited high genetic diversity

The 124 *L*. *(V*.*) braziliensis* strains exhibited one or two peaks in all electropherograms analyzed. GenAlEx 6.5 determined that the 124 strains of *L*. *(V*.*) braziliensis* exhibited a high number of alleles per locus with a mean allelic diversity of 14.9, ranging from 6 to 26 alleles per locus (markers Ibh3 and AC52, respectively). Also, the microsatellite markers analyzed in the total *L*. *(V*.*) braziliensis* population showed high average expected heterozygosity (*H*_*E*_ = 0.816), slightly higher than observed heterozygosity (*H*_*O*_ = 0.669), high mean polymorphic information content (PIC = 0.790) (Table 2), and a mean inbreeding coefficient of 0.18. Genepop v4.4 analysis showed a significant deviation from Hardy-Weinberg equilibrium for the observed heterozygote deficit in the majority of markers, except for ARP and G09 (p > 0.05) in the total population (124 strains).

**Table 2:** Genetic diversity parameters of the 10 *L*. *(V*.*) braziliensis* microsatellite loci.

Locus	*N*_*A*_	*H*_*O*_	*H*_*E*_	*PIC*
**AC52**	26	0.831	0.936	0.928
**B3H**	18	0.553	0.835	0.812
**Ibh3**	6	0.266	0.556	0.506
**E11**	9	0.577	0.739	0.702
**7GN**	13	0.734	0.853	0.833
**EMI**	13	0.748	0.829	0.804
**LBA**	15	0.806	0.904	0.892
**ARP**	20	0.855	0.922	0.913
**G09**	9	0.542	0.664	0.601
**LRC**	20	0.782	0.923	0.913
**Mean**	**14.9**	**0.669**	**0.816**	**0.790**

*N*_*A*_ = allelic diversity, *H*_*O*_ = observed heterozygosity, *H*_*E*_ = expected heterozygosity, *PIC* = polymorphic information content. *N*_*A*_, *H*_*O*_, and *H_E_* were estimated with GenAlEx 6.5; PIC was estimated with CERVUS 3.0.

On the other hand, genetic diversity parameters showed that strains collected in the lowland jungle had more variability than those collected in the highland jungle when considered the pre-established ecoregions (Table 3).

**Table 3:** Genetic diversity for each pre-established ecoregion.

*Ecoregion*	*Strains*		*N*_*A*_	*H*_*O*_	*H*_*E*_	*F*_*IS*_
**Highland jungle**	25	Mean	3.400	0.329	0.386	0.169
		SD	0.670	0.080	0.091	-
**Lowland jungle**	99	Mean	14.400	0.753	0.781	0.041
		SD	1.968	0.058	0.057	-

All parameters were estimated with GenAlEx 6.5.

However, AMOVA analysis showed that most percentages of the variation were between individuals within populations (82.15%) rather than by pre-established groups or ecoregions (9.39%) (Table 4).

**Table 4:** Analysis of Molecular Variance (AMOVA) for *L*. *(V*.*) braziliensis* population.

*Source of variation*	*Sum of squares*	*Variance components*	*Estimated variation (%)*
Among ecoregions	74.41	0.42	9.39
Among departments within ecoregions	40.66	0.38	8.46
Between individuals within populations	887.76	3.69	82.15
Total	1002.84	4.49	100.00

Results were determined with Arlequin 3.5.

### The population structure of *L*. *(V*.*) braziliensis* showed a marked differentiation by ecoregions

**Fig 2:**
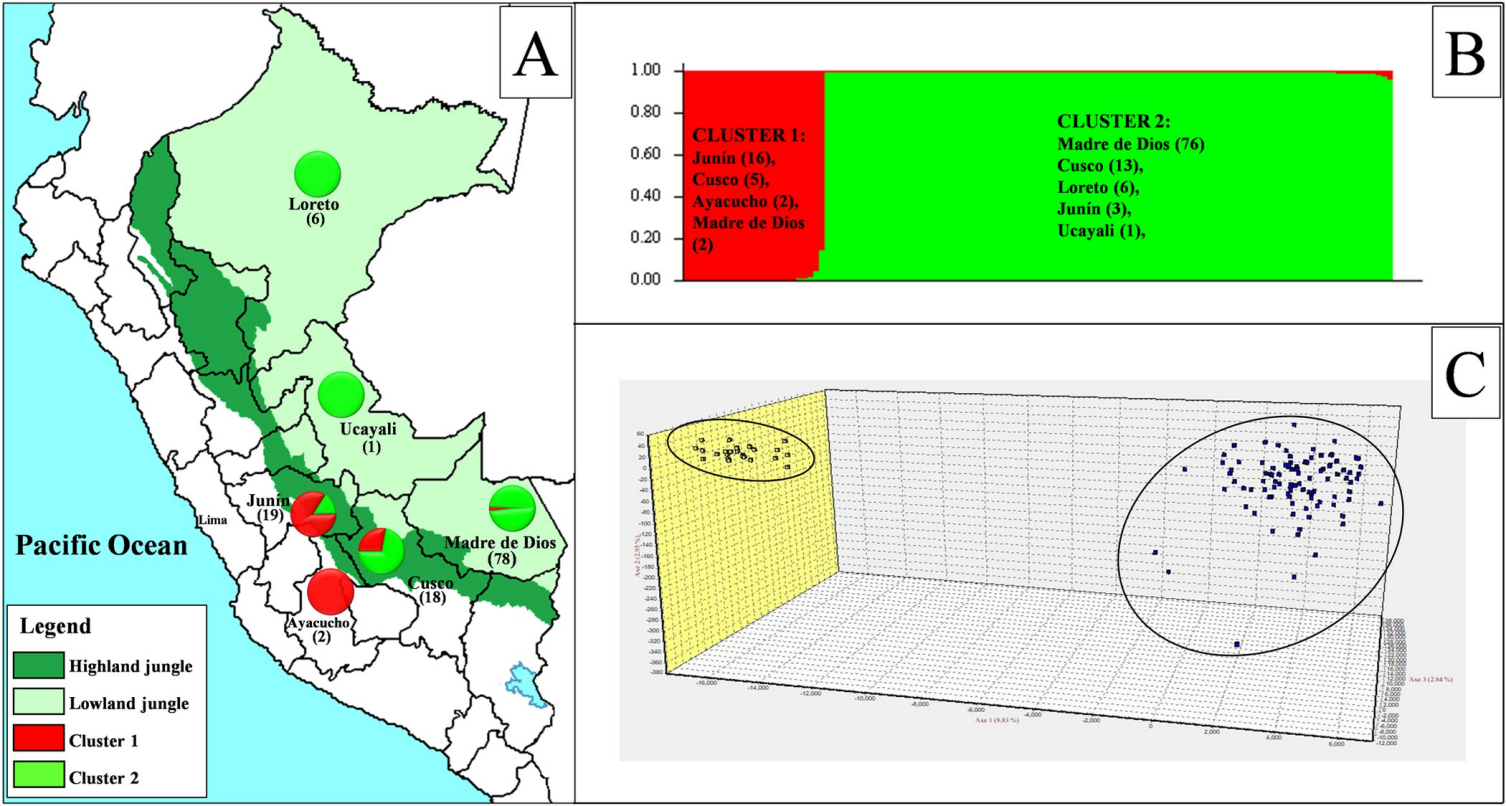
Presence of two *L*. *(V*.*) braziliensis* genetic clusters from the Peruvian jungle. (**A)** Map of Peru showing the geographical ecoregions where the highland jungle in dark green is represented by samples from the departments of Junín, Cusco, and Ayacucho, and lowland jungle in light green includes samples from Loreto, Ucayali, and Madre de Dios. The pie charts describe the distribution of the two subpopulations inferred where Cluster 1 is in red and Cluster 2 is in green. (The map is based on a public map downloaded from https://d-maps.com/carte.php?num_car=4765&lang=en). **(B)** Graphic of STRUCTURE v2.3 analysis showing the number of strains isolated by each department with 25 strains for Cluster 1 and 99 strains for Cluster 2. **(C)** Factorial correspondence analysis (FCA) plot where 25 yellow squares correspond to strains of cluster 1 and 99 dark blue squares represent Cluster 2.

The 124 *L*. *(V*.*) braziliensis* strains were grouped into two genetic clusters by STRUCTURE v2.3 (S3 Table and S1 Fig). Cluster 1 comprised 25 strains, from which 23 were isolated from the highland jungle of the departments of Junín, Cusco, and Ayacucho and two strains from Madre de Dios in the lowland region (Fig 2A and 2B, in red).

Cluster 2 comprised 99 strains, 83 of these were isolated from the lowland jungle of the departments of Loreto, Ucayali, and Madre de Dios, and 16 strains were from Cusco and Junín (Fig 2A and 2B, in green). The factorial correspondence analysis (FCA) showed a similar grouping (Fig 2C). GenAlEx determined private alleles or alleles unique to a single population; it reported 0.5±0.2 alleles for highland jungle and 11.5±1.8 alleles for lowland jungle. Fixation indices *F*_*ST*_ (0.319) and *Rho*_*ST*_ (0.434) showed moderately high genetic differentiation in the two clusters found (S4 Table). In addition, the analysis of the Neighbor-Joining tree confirmed the results showed by STRUCTURE and FCA where Cluster 1 groups 25 strains from Junín, Cusco, and Ayacucho, while Cluster 2 groups 99 strains mainly from Madre de Dios, Loreto, and Ucayali (S2 Fig).

Given the different sample sizes between clusters, the allelic richness analysis after the rarefaction method was based on a sample size of 21 diploid individuals per cluster, which was the smallest number of individuals to be analyzed (Table 5).

**Table 5:** Allelic richness for both clusters.

Marker	Cluster 1	Cluster 2
AC52	2.977	18.387
B3H	1.000	10.586
Ibh3	1.000	3.281
E11	2.840	5.543
7GN	3.000	10.345
EMI	4.840	9.847
LBA	3.000	12.451
ARP	7.634	15.164
G09	2.000	5.637
LRC	4.677	13.722

The results were determined with FSTAT 2.9.3.2.

### Characterization of subpopulations in Peruvian *L*. *(V*.*) braziliensis*

The mean inbreeding coefficient *F*_*IS*_ values were 0.1693 (95% CI 0.0118–0.3085) and 0.0413 (95%CI 0.0204–0.0696) for Clusters 1 and 2, respectively (Table 3).

In addition, pairwise linkage disequilibrium analysis presented 11.1% (5 pairs of loci from 45) and 17.8% (8 pairs of loci from 45) of non-random association between loci after Bonferroni correction (p < 0.001) in Cluster 1 and 2, respectively. Genetic Cluster 1 exhibited standardized index of association, $\bar{r_{d}}$ values of 0.060 (p = 0.000) and the Cluster 2, 0.001 (p = 0.429) in the multilocus analysis.

### Possible event of colonization in *L*. *(V*.*) braziliensis*

Two geographic units were characterized: the Peruvian highland jungle and lowland jungle. For the statistical analysis, geographic units were treated as two populations, all possible connection models between these two populations were evaluated with MIGRATE-N v4.4.4; these models can be grouped into 1) models of ongoing immigration, 2) models of colonization with migration after the colonization event, and 3) models of colonization without immigration after the colonization event. All models with immigration have model probabilities of 0.0000 and low log marginal likelihoods. Only the model where the highland is colonized from the lowland has high model probability (the models 3b and 3c specify the same model but differ in their prior probability range: 3c investigated events only in the colonization time range of 0 to 50, whereas 3b used a range of 0 to 100 (Table 6). Hence, the occurrence of *L. (V.) braziliensis* in the highlands is likely a result of a colonization event from the lowlands.

**Table 6:** Probability of models proposed to analyze possible events of gene flow and colonization.

*Model*	*Log(marginal Likelihood)*	*LBF*	*Model-probability*
1a:	-29428.2	-3611.72	0
1b:	-28569.2	-2752.72	0
1c:	-28291.15	-2474.67	0
2a:	-27422.57	-1606.09	0
2b:	-26910.43	-1093.95	0
2c:	-26866.04	-1049.56	0
2d:	-26714.12	-897.64	0
3a:	-26097.23	-280.75	0
3b:	-25816.48	0	0.9998 (*)
3c:	-25824.81	-8.33	0.0002 (*)

LBF = Log Bayes Factor.

*Difference between models 3b and 3c was a uniform prior probability of 0 to 100 and 0 to 50 generation × mutation rate, respectively.

Assuming that the parasite lives in the vector insect with a replication time of 7.6 hours and replicates in human hosts every 48.8 hours [38], and based on a wide range of mutation rates for microsatellites [39], we estimate that the limits for the colonization event, in years, were more recent in vectors than in human hosts (S1 File and S5 Table).

## Discussion

This study confirms the high genetic diversity in *L*. *(V*.*) braziliensis* from the Peruvian Amazon rainforest with two clusters distributed across the highland jungle, *Rupa-Rupa*, and lowland jungle, *Omagua* [14]. Furthermore, it reveals a possible event of past colonization of the highland jungle from the lowland jungle without subsequent gene flow.

The quality and quantity of DNA isolated from *in-vitro* cultured *Leishmania* promastigotes were crucial for the successful amplification of the ten polymorphic microsatellite markers by PCR. Previous assays that we performed with DNA extracted from other types of clinical samples like biopsies, lancet scrapping, and filter paper imprints resulted in poor resolution of certain microsatellite markers due to the presence of artifacts, stutters principally, and absence of amplification which made it challenging to identify the true alleles [40].

Most of the ten markers showed new alleles reflecting high polymorphism in the populations of the parasites isolated from different Peruvian regions compared to the range of alleles previously reported (see S6 Table) [5,6]. The finding of high genetic diversity (*N*_*A*_ = 14.9, *H*_*E*_ = 0.816) is consistent with previous studies conducted in Peru. One of them with 124 *L*. *(V*.*) braziliensis* strains from different countries including 25 strains from the endemic zone of Pilcopata, Cusco showed *N*_*A*_ = 12.4 and *H*_*E*_ = 0.75 [6]. A second study assessed 56 *L*. *(V*.*) braziliensis* strains from an unknown location in Peru and reported *N*_*A*_ = 9.7 and *H*_*E*_ = 0.77 [19]. Our study included isolates from the mountainous or highland jungle and swampy or lowland jungle from central and southeast Peru, specifically from endemic regions of Madre de Dios (78 strains) and Cusco (18 strains) adding evidence to published studies about genetic variability in this parasite [6,9,19].

The high genetic diversity that we observe could be explained in part by the type of reproduction of the parasite. Interestingly, both clusters have moderate *F*_*IS*_ values, being *F*_*IS*_ value for Cluster 2 (*F*_*IS*_ = 0.041, associated with lowland jungle) near zero that could suggest high recombination. This result is similar to the high recombination rates suggested for *L*. *(V*.*) braziliensis* from different regions of Peru, using whole-genome sequencing (WGS, 21 isolates, mean *F*_*IS*_ = -0.11) [10]. In contrast, Cluster 1 (*F*_*IS*_ = 0.169, associated with highland jungle) has a higher *F*_*IS*_ value than Cluster 2, suggesting a more recent establishment due to migration or colonization. Our *F*_*IS*_ values are lower than those found in Pilcopata, Cusco, Peru (25 isolates in 1993–1994 with *F*_*IS*_ = 0.5) and Acre, Brazil (63 isolates from 1980 to 2008 with *F*_*IS*_ *=* 0.519) [6,8]. These two studies had limited samples per geographic region and strong population subdivision (Wahlund effect), suggesting partially clonal populations. Similarly, to eliminate space-time bias leading to a Wahlund effect in our study, more samples should have been collected from other Peruvian regions, but only low numbers of annual cases were reported during years of our collection (2012–2016) compared to higher cases we found in endemic regions of Cusco and Madre de Dios [41]. Also, the linkage disequilibrium between pairs of loci and multilocus for both Peruvian subpopulations of *L*. *(V*.*) braziliensis* was not strong, suggesting that the reproduction could not be strictly clonal similar to the previous report in Brazil [8]. Interspecific genetic recombination or the presence of hybrids in contrast to earlier reports have not been found in our study [42,43].

Although the genetic clusters described are distributed between the ecoregions of highland and lowland jungles, most isolates are from three areas: Junín, Cusco, and Madre de Dios. A deeper analysis indicates significant differences in geographic variation. In particular, the samples from the districts of Tahuamanú, Las Piedras, and Tambopata in Madre de Dios show high variability as a result of the intrapopulation contribution to the diversity found by AMOVA. The higher allelic richness in cluster 2 reinforces the high diversity observed principally in the *Omagua* and part of *Rupa-Rupa* regions, compared with the diversity of alleles grouped in Cluster 1 present mainly in the highland jungle. A possible explanation for the extensive diversity may be the presence of several hydrographic basins in Madre de Dios facilitating isolation and genetic variation of the parasite. However, the rivers and altitudinal floors do not seem to be an unbeatable barrier for the dispersal of possible vectors of the subpopulations of *L*. *(V*.*) braziliensis* [44]. Recently we have described the presence of some putative vector species (*Lutzomyia auraensis*, *Lu*. *davisi*) naturally infected with *L*. *(V*.*) braziliensis* in Madre de Dios [45,46]. The same sandfly species were found infected with *L*. *(V*.*) braziliensis* in the Brazilian state of Acre, neighbor to Madre de Dios [47,48]. Remarkably, the two clusters identified here do not appear in an earlier work where the authors, through WGS, focused on the speciation and diversification of *L*. *(V*.*) peruviana* and *L*. *(V*.*) braziliensis*. Their temporal and spatial sampling scheme [10] differs from our study. They collected isolates from 1990 to 2003; most isolates were from Cajamarca, Huánuco, Pasco, and collection altitudes were around 630 meters and higher than 1900 meters. In contrast, our isolates were from 2012 to 2016 and principally from geographical areas lower than 1000 meters from Junín, Cusco, and Madre de Dios. This study [10] suggested high gene flow in contrast with the marked genetic differentiation that we found between the two clusters and the MIGRATE-N results that showed colonization of *L*. *(V*.*) braziliensis* from lowland jungle to highland jungle and posterior populational isolation without gene flow. Discrepancies could derive from differences in the number of representative strain isolates, site of sampling, and methodological approaches used, MLMT versus WGS.

Cluster 2 distributed between the Peruvian lowland jungle and part of the highland jungle could be similar to subpopulation 3 of a previous study that grouped six strains of *L*. *(V*.*) braziliensis* from the mountainous region and lowland jungle of Peru with two strains from the neighboring Brazilian state of Acre, using 15 MLMT loci [5]. Two of those markers were assessed in our study (B3H and 7GN) considering high number of alleles shown. Likewise, Cluster 2 could be similar to subpopulation Pop3A that grouped strains of the Brazilian state of Acre close to the border with Madre de Dios [8].

The observation that some alleles from the lowland jungle were present in the highland jungle, despite the STRUCTURE, FCA, and Neighbor-Joining tree findings of two completely separate *L*. *(V*.*) braziliensis* genetic groups, led us to search for an explanation. We assumed several different models of gene flow and colonization between the altitudinal regions. The role of human migration was investigated because cutaneous and mucosal leishmaniasis are zoonotic diseases without the involvement of humans in the transmission [49]. In addition, patients from whom parasites were isolated performed different activities: military deployed in the highland jungle (Junín, Cusco, Ayacucho) and civilians living in the lowland jungle (Loreto, Ucayali, Madre de Dios) without contact with each other at the time of sampling. Likewise, known human migration from Cusco and Puno to Madre de Dios through the Interoceanic Highway [50] was dismissed because no contact was observed between both groups of people and no register of possible migration between civilians. The recent colonization event could have been driven by the participation of the vectors and the reservoirs in historical times [46,51–53]. Spanish chroniclers and studies on human skulls reported human migration from the highland jungle to the western Andean valleys [54–56]. More recent human migration from other Peruvian departments to Cusco and Madre de Dios due to economic factors [57] would partially explain the possible recent colonization event, before the time of our study. We do not rule out that patients during written informed consent may give inaccurate data about the geographic location where possibly they became infected. Therefore, the infection may have occurred before they were to work in the highland jungle (military) or in the lowland jungle (civilian population), where they eventually developed the disease.

As mentioned above, the MIGRATE-N results and divergence time in coalescent units showing a recent colonization event from lowland jungle to highland jungle could explain the geographic distribution of Cluster 2 in both regions (S1 File and S5 Table). This event is more recent than the speciation and diversification of the *L*. *(V*.*) braziliensis* complex in the late Pleistocene [10].

No correlation was found between clinical characteristics from patients (size of lesion, location of lesion, number of lesions, time of disease, age of patients, response to treatment) and a particular parasite genetic profile, similar to that already described by many studies [8,58]. Other factors such as the nutritional and immunological status of the patient may explain the differences in disease outcome [8,59,60]. Likewise, different parasite and vector factors explain the pleiomorphism of leishmaniasis [61] that cannot be explained only by the analysis of genetic diversity and population structure described. It is expected that studies applying whole-genome sequencing of *Leishmania* can provide epidemiological information to design control strategies in endemic regions [62,63].

The high genetic diversity and population structure of *L*. *(V*.*) braziliensis* described in our work will help better understand the evolutionary history of this parasite in Peru [9,10,64,65]. New epidemiological studies may benefit from the knowledge of the distribution of the leishmaniasis agent [66,67] to develop transmission mitigation strategies between military [68,69] and civilian people in endemic areas of Peru [70,71].

## Supporting information

S1 Fig Delta K (ΔK) and K values inferred by Structure Harvester. (TIF)

S2 Fig Neighbor-Joining tree, radiated style, shows the two clusters for *L*. *(V*.*) braziliensis*. The genetic distance was measured by Nei’s minimum distance.
(TIF)

S1 Table Sample code, geographic origin, year of enrollment and all genotypes obtained for 10 microsatellite markers. ^1^Precise sizes of amplified fragments that represent genotypes obtained at each of 10 loci by PCR. NA, fragment non-amplified.
(XLSX)

S2 Table Micro-Checker results for stuttering, allele dropout and null alleles. A) Values for all populations. B) Values for cluster 1, 25 isolates. C) Values for cluster 2, 99 isolates.
(TIF)

S3 Table The Evanno table output with K values obtained by STRUCTURE. (TIF)

S4 Table Values of *F*_*ST*_ and *Rho*_*ST*_ obtained with Weir and Cockerham method in Genepop 4.0.10. (XLSX)

S5 Table Time of colonization of the highland from the lowland jungle. Multiple potential translations of the divergence time estimate of MIGRATE are given: g, generation; μ, locus mutation rate per generation; the 2.5% and the 97.5% are percentiles of the posterior distribution of the divergence time and mark the boundary of the 95% confidence interval. The microsatellite mutation rate values are not known for *Leishmania* but are probable values [39]. The generations per year were calculated from replication times in promastigotes and amastigotes [38].
(XLSX)

S6 Table Comparison of the range and distribution of alleles reported with the described in this study. ^**1**^Range of allele size reported for 124 strains of *L*. *(V*.*) braziliensis* in reference [6] where 25 strains were from Peru. ^2^Range of fragment size obtained for 31 strains of *L*. *(V*.*) braziliensis* in reference [5], where six strains were from Peru. ^3^Range of fragment size reported for 21 Peruvian strains of *L*. *(V*.*) braziliensis* in reference [7]. ^4^Range of fragment size found in this study for 124 Peruvian strains of *L*. *(V*.*) braziliensis*.
(XLSX)

S1 File Calculation of the time of colonization. (DOCX)
