# Supplementary figures and images for: Genetic diversity and population structure of Leishmania (Viannia) braziliensis in the Peruvian jungle
Source: PLoS Negl Trop Dis. 2022 May 23;16(5):e0010374. doi: 10.1371/journal.pntd.0010374 (PMC9126394; doi:10.1371/journal.pntd.0010374)

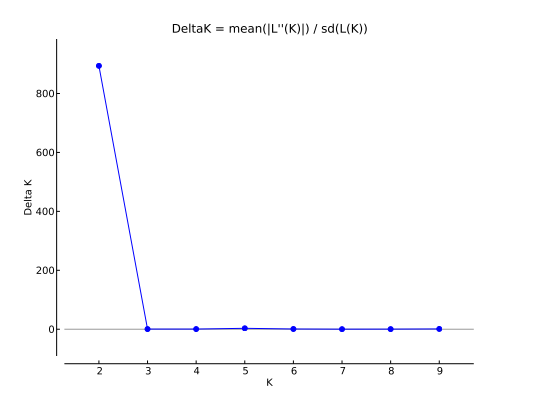

Supplement: S1 Fig — (TIF) [file pntd.0010374.s001.tif]

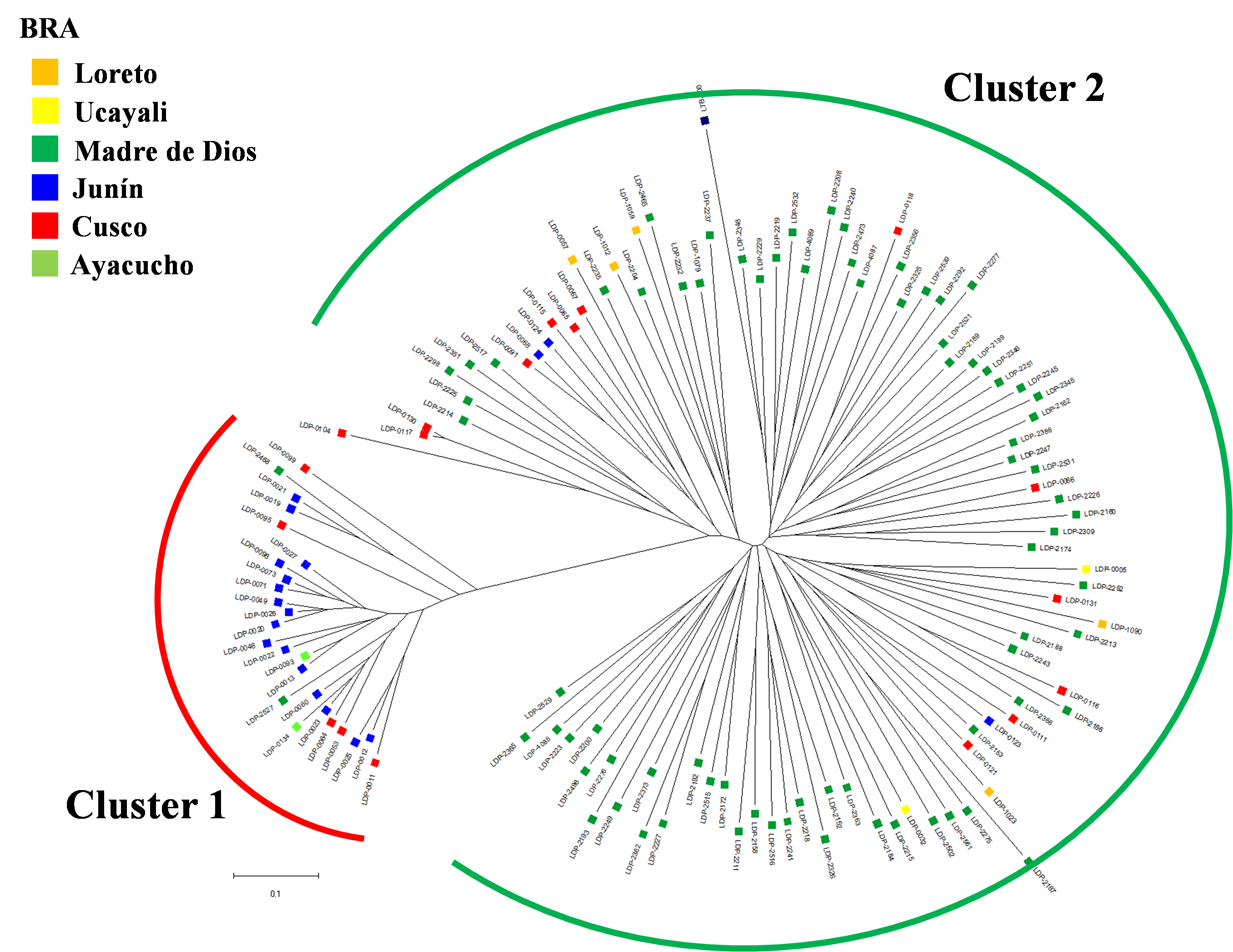

Supplement: S2 Fig — The genetic distance was measured by Nei’s minimum distance. (TIF) [file pntd.0010374.s002.tif]

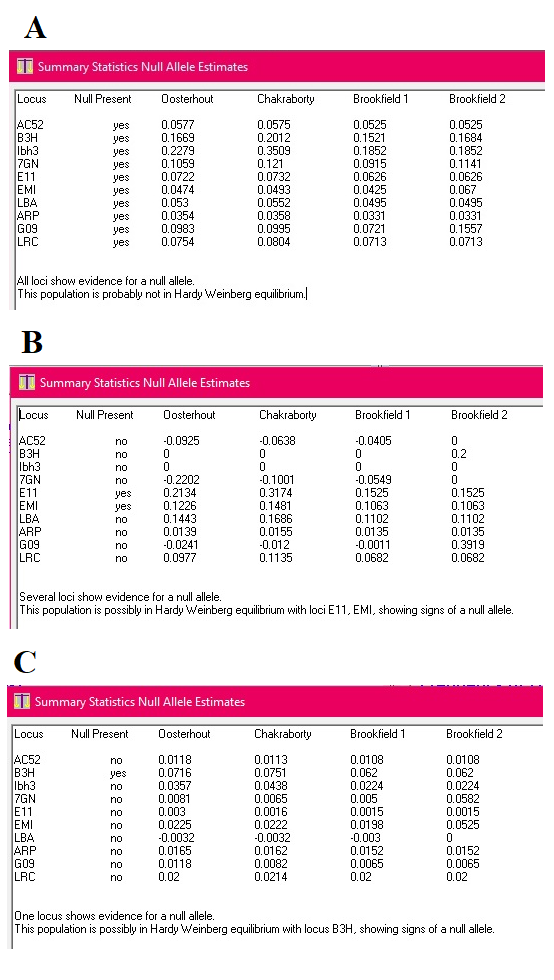

Supplement: S2 Table — A) Values for all populations. B) Values for cluster 1, 25 isolates. C) Values for cluster 2, 99 isolates. (TIF) [file pntd.0010374.s004.tif]

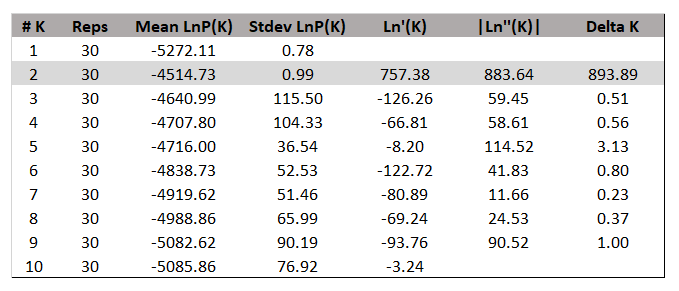

Supplement: S3 Table — (TIF) [file pntd.0010374.s005.tif]
