# Supplementary material for: Genetic diversity and population structure of Leishmania (Viannia) braziliensis in the Peruvian jungle
Source: PLoS Negl Trop Dis. 2022 May 23;16(5):e0010374. doi: 10.1371/journal.pntd.0010374 (PMC9126394; doi:10.1371/journal.pntd.0010374)
Supplement: S1 File — (DOCX) [file pntd.0010374.s009.docx]

**S1 File.**

We extend the results with a table that shows the range of estimated colonization times for the term "recent event". Using the replication time of 7.6 hours in sandflies and 48.8 in humans [1] as a measure for the rotation of generations, we obtain in sandflies 3.158 generations per day, then 1152.6 generations in a year. In contrast, with slow replication in humans, we obtain 0.492 generations per day, 179.5 generations in a year. If we also assume a wide mutation rate range for microsatellites between 10 ^-4^ and 10 ^-6^ [2], then we obtain limits for the colonization event in sandflies between ~3 years and ~1570 years. If we assume that *Leishmania* replicates in humans, then we reach limits of ~18 years and ~10,000 years. For comparisons with other species, the divergence time in coalescent units certainly shows that the colonization was recent, given the available population size of *Leishmania*.

**References**

1. Jara M, Maes I, Imamura H, Domagalska MA, Dujardin JC, Arevalo J. Tracking of quiescence in *Leishmania* by quantifying the expression of GFP in the ribosomal DNA locus. Sci Rep. 2019;9. doi:10.1038/s41598-019-55486-z

2. Seyfert AL, Cristescu MEA, Frisse L, Schaack S, Thomas WK, Lynch M. The rate and spectrum of microsatellite mutation in *Caenorhabditis elegans* and *Daphnia pulex*. Genetics. 2008;178. doi:10.1534/genetics.107.081927
